# Male mammary gland development after apatinib therapy in advanced gastric cancer: A case report

**DOI:** 10.1097/MD.0000000000020727

**Published:** 2020-07-10

**Authors:** Yan Hui, Jingchun Wang, Jianguo Han, Zhifeng Guo, Siqi Sun, Zifang Wu, Yanqiu Wang, Zhimin Han, Xiangbin Chen

**Affiliations:** a Chifeng Municipal Hospital, Inner Mongolia; b Geneis Beijing Co. Ltd., Beijing, China.

**Keywords:** adverse reactions, apatinib, gastric cancer, male mammary gland development

## Abstract

**Rationale:**

Most gastric cancer patients are diagnosed at mid- to late-stage and lose the chance of radical surgery, medical treatment is especially important to prolong the survival of patients. Apatinib mesylate, which is a small molecule vascular endothelial growth factor receptor 2 tyrosine kinase inhibitor, could be used as antiangiogenesis therapy for gastric cancer.

**Patient concerns:**

A 67-year-old man sought medical care for upper abdominal discomfort.

**Diagnosis:**

The patient was diagnosed as mixed medullary differentiated gastric adenocarcinoma, and immunohistochemistry suggested HER-2 (2+).

**Interventions:**

The patient received chemotherapy consisting of oxaliplatin combined with S-1 as first-line treatment, and targeted therapy with apatinib mesylate as second-line treatment.

**Outcomes:**

After 4 months of first-line chemotherapy, the patient received apatinib treatment immediately at a dose of 500 mg/d orally and died of cardiac arrest with 8.5 months of overall survival. During this period of targeted therapy with apatinib mesylate, this male patient suffered mammary gland development besides other common adverse reactions.

**Lessons:**

This case report is the first to report the case of male mammary gland development after oral apatinib.

## 1 Introduction

Since most gastric cancer patients lose the chance of radical surgery when diagnosed at middle or late stage, medical treatment plays crucial roles in prolonged survival of patients.^[1,2]^ Apatinib mesylate blocks signal transduction by binding of vascular endothelial growth factor (VEGF) to its receptor, and could be used as antiangiogenesis therapy for multiple cancers.^[3]^ Although common adverse reactions of apatinib include hypertension, proteinuria, hand-foot syndrome, gastrointestinal bleeding, etc, so far there has been no reported case of male mammary gland development after oral apatinib. Here we report a case of a male gastric cancer patient suffered mammary gland development besides above-mentioned common adverse reactions of apatinib after 1month of treatment. To our knowledge, this is the first report on male mammary gland development caused by apatinib.

## 2 Case presentation

A 67-year-old man sought medical care for upper abdominal discomfort in December 2016. Gastroscopy revealed gastric-to-gastric antrum lesions. Pathological examination suggested mixed medullary differentiated adenocarcinoma, and immunohistochemistry suggested HER-2(2+). On February 20, 2017, radical gastrectomy was performed. The tumor had invaded the main body of the pancreas, so palliative gastrojejunostomy was selected. Cancer cells were suspected in the postoperative peritoneal lavage fluid.

### 2.1 First-line treatment

The patient was given systemic chemotherapy (March 30, 2017) consisting of oxaliplatin combined with S-1, and he declined detection of HER-2 FISH for personal reasons; III-degree myelosuppression occurred during treatment, manifesting as a decrease in white blood cells and platelet count. Oral S-1 was ceased 12 days later. On April 28, May 23, and June 19, 2017, the chemotherapeutic dose was decreased to 75%, II-degree myelosuppression occurred, and bone marrow suppression recovered slowly. On September 9, abdominal computed tomography was performed: the right posterior lobe of the liver showed a low-density lesion 4.5×2.1 cm in size, and the enhanced scan showed a ring-shaped enhancement, suggesting a metastatic tumor. The assessment was for progressive disease (liver metastasis) with a progression-free survival of 4 months.

### 2.2 Second-line treatment

**Figure 1:**
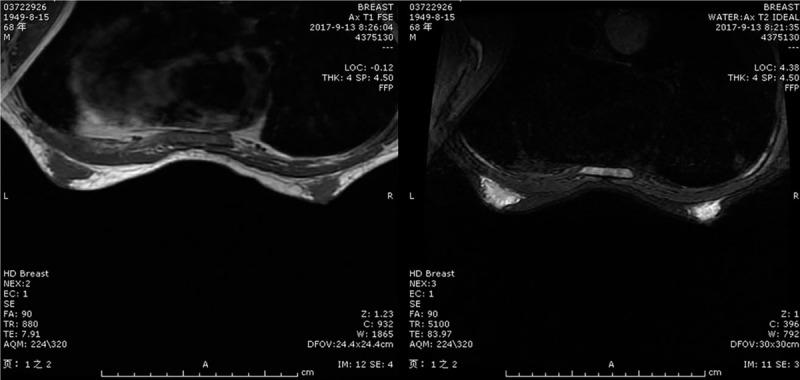
Magnetic resonance imaging T1 and T2 weighted images show developmental mammary gland images.

Considering it is still difficult to recover bone marrow suppression after reducing the dose of chemotherapy this patient previously received, targeted therapy was selected for the second-line treatment. The targeted therapy was apatinib mesylate tablets at an initial dose of 500 mg/d (August 10 through October 20, 2017); 1 month after the beginning of treatment (September 13, 2017), a review of abdominal computed tomography findings showed gastric cancer, the range of postoperative gastrojejunostomy was relative stable, and the range of low density lesion of the right-posterior lobe of the liver was reduced to 3.9 × 1.5 cm; the efficacy was classified as partial remission according to the RESIST 1.0 standard. The patient's conscious bilateral breast pain was obvious on September 10, and the bilateral mammary gland was palpable, hard lumps, and tenderness. Male mammary gland development was diagnosed according to the breast ultrasound and breast magnetic resonance imaging results (Fig. 1), and the left and right ranges were 2.3 × 0.9 cm, 1.5 × 0.7 cm, respectively. The patient reported skin itching; urine routine prompts urine protein (3+); blood routine tips for white blood cells 2.0 × 10^9^/L, platelets 74 × 10^9^/L; and thickening of the stratum corneum at the finger joints with mild pain, though he had no trouble with normal physical activity. The patient's blood pressure was 150/100 mm Hg; apatinib was stopped. The patient was given auxiliary hematopoietic and antihypertensive symptomatic treatment and then reexamined. Urine routine analysis showed urine protein (–), blood routine results returned to normal, and blood pressure was controlled under 130/80 mm Hg. The breast ultrasound result showed male mammary gland development. Oral apatinib was continued, and the dose was kept at 500 mg/d because the patient continued to tolerate it. On October 20, routine urine analysis showed urinary protein 2+, and the patient produced black stool, suggesting upper gastrointestinal bleeding. Oral apatinib tablets were ceased. On November 2, the patient suddenly had a cardiac arrest and could not be resuscitated. As of the patient's death, second-line treatment progression-free survival had lasted 2.3 months, and his overall survival was 8.5 months.

## 3 Discussion

Apatinib mesylate is a small molecule vascular endothelial growth factor receptor 2 (VEGFR-2) tyrosine kinase inhibitor that blocks signal transduction by binding of vascular endothelial growth factor (VEGF) to its receptor, potently inhibits tumor angiogenesis, and plays an anti-tumor therapeutic role.^[3,4]^

Due to his history of high blood pressure, the patient showed hand-foot syndrome, proteinuria, itchy skin, and continued high blood pressure after 1 month of apatinib treatment. During this period, proteinuria 3+ occurred, and recovered after 10 days of stopping apatinib treatment; the patient's skin was itchy, and no drug intervention was given because the side effects were mild. Unlike other patients, this patient developed the above-mentioned adverse reactions and developed male mammary gland development. Previous studies have shown that targeted anti-tumor drugs such as imatinib could induce male mammary gland development,^[5,6]^ specifically, 18% (7/38) of patients who received imatinib treatment had developed male mammary gland development in that study.^[6]^ Moreover, it was suggested the mechanism of imatinib inducing male mammary gland development was blocking testosterone synthesis involved in tyrosine kinases.^[5,6]^ Another case reported that kidney cancer patients who developed male gynecomastia under treatment of sunitinib, which is a multi-target receptor tyrosine kinase inhibitor, including VEGF.^[7]^ An experimental study used mice suggested that neutralizing VEGF bioactivity also reduced the testosterone-stimulated growth of the glandular epithelial tissue. This effect, as the previously demonstrated effect of anti-VEGF treatment on corpus luteum growth, is secondary to inhibition of vascular growth.^[8]^ However, we did not check the patient's testosterone levels which may provide more clues to our study. Since apatinib mesylate is also a tyrosine kinase inhibitor, it is speculated the use of this drug could be the cause of male mammary gland development in our study. As there has been few study reported in this area yet, more research would be need to verify.

## 4 Consent

We have obtained written informed consent from the patient's daughter for publication of the patient's details.

## Author contributions

**Conceptualization:** Yan Hui.

**Data curation:** Jingchun Wang, Jianguo Han, Zhifeng Guo, Siqi Sun, Zifang Wu, Yanqiu Wang, Zhimin Han.

**Writing – original draft:** Xiangbin Chen.
